# Alcoholic Beverage Consumption in India, Mexico, and Nigeria: A Cross-Cultural Comparison

**Published:** 1998

**Authors:** Linda A. Bennett, Carlos Campillo, C.R. Chandrashekar, Oye Gureje

**Affiliations:** Linda A. Bennett, Ph.D., is an associate dean at the College of Arts and Sciences and professor of anthropology, University of Memphis, Memphis, Tennessee. Carlos Campillo, M.D., is project coordinator in the Department of Epidemiology and Social Research in the Instituto Mexicano de Psiquiatria (IMP), University of Mexico, Mexico City, Mexico. C.R. Chandrashekar, M.D., is additional professor of psychiatry at the National Institute of Mental Health and Neuro Sciences, Deemed University, Bangalore, India. Oye Gureje, M.B.B.S., Ph.D., FRANCZP, is a professor in the Department of Psychiatry, University of Ibadan, Ibadan, Nigeria

**Keywords:** AOD consumption, South Central Asia, western Africa, Mexico, cross cultural study, comparative study, ethnic differences, international differences, AOD preference, gender differences, age differences, context dynamics, AOD use pattern, problematic AOD use, epidemiology, public policy on AOD use, attitude toward AOD, AOD prevention, treatment, literature review

## Abstract

Drinking practices vary substantially among different countries. An understanding of such differences can help researchers, clinicians, and policymakers develop prevention, diagnostic, and treatment measures as well as overall alcohol policies that are appropriate for a given country. Accordingly, researchers have conducted cross-cultural analyses of drinking patterns and practices. Three countries included in such analyses are India, Mexico, and Nigeria. These countries differ substantially in their ethnic and cultural characteristics, including the role that alcohol plays in daily life. To gain a better insight into the attitudes toward alcohol in these countries, researchers have analyzed the alcoholic beverage preferences, gender and age differences in alcohol consumption patterns, drinking contexts and drinking patterns, alcohol-related problems, approaches to prevention and treatment, and drinking indicators in each nation. These analyses demonstrate that no single definition of “normal” drinking, problem drinking, or alcohol dependence can apply equally to all countries or cultures.

Alcoholic beverage consumption patterns vary considerably among different countries and even among different ethnic groups within one country. These variations in drinking patterns include, for example, the types of beverages consumed preferentially, occasions on which consumption typically occurs, drinking levels that are considered normal, and population subgroups for whom drinking is considered acceptable. Television, movies, and scholarly publications have depicted and investigated differences in drinking traditions to such an extent that people throughout the world are increasingly aware of drinking patterns in cultures other than their own.

Several decades ago, researchers in the United States began paying considerable attention to the role that alcohol consumption plays in the lives of people of different ethnic backgrounds. Such investigations initially focused on the differences among people of Irish, Italian, and Jewish descent living in the United States. More recently, interest has focused on the drinking patterns among various Native American, African-American, and Hispanic groups. Similar studies on the diversity of drinking traditions have been conducted in Europe, comparing, for example, drinking patterns between Mediterranean and Scandinavian countries. In addition, studies conducted in South and East Asia have demonstrated that the roles of alcohol consumption in daily life and on ritual occasions vary considerably among different countries or cultures and depend in part on the religious practices of the groups studied.

What is the relevance of studies on cross-cultural differences in alcoholic beverage consumption? Cultural variations in drinking practices and beliefs about alcohol have far-reaching implications for policymakers, clinicians, and researchers in the field of alcohol and other drug (AOD) use and abuse. Based on cross-cultural comparisons, combined with information on historical changes and variations in drinking behavior within each culture, researchers and policymakers can obtain a better understanding of the relationships between drinking patterns, drinking-related consequences, and the outcome of informal and formal alcohol policies. Such analyses can assist in the development of appropriate alcohol-related policies and in the identification of effective clinical prevention and treatment strategies. For example, during the early 1980s the Institute of Applied Social and Economic Research (IASER) in Papua New Guinea conducted a cross-cultural examination of traditional drinking practices and the effects of deprohibition. The results of that evaluation were used to assist the development of public policies on drinking (Marshall 1982).

This article compares the drinking practices of people living in India, Mexico, and Nigeria. Each of these three countries has a highly diverse and multicultural population, and major differences exist between their urban and rural areas. Nevertheless, the three countries studied differ sharply in drinking practices. These differences allow for the exploration of potential implications of cultural variations in drinking patterns for the identification, prevention, and treatment of drinking-related problems currently encountered in India, Mexico, and Nigeria. Following a brief description of the cultural and ethnic characteristics, this article reviews each country’s alcoholic beverage preferences, epidemiological patterns in alcohol consumption, typical drinking contexts and cultural drinking norms, alcohol-related problems, and approaches to prevention or treatment.

## Cultural and Ethnic Characteristics of the Study Regions

India, Mexico, and Nigeria were selected for cross-cultural comparisons in this article because they represent areas with distinctly different geographic locations and predominant languages and religions (see table 1). Moreover, the three countries have distinct histories of traditional alcohol consumption as well as widely contrasting contemporary drinking patterns and alcohol-related problems. All three countries participated in the Cross-Cultural Applicability Research (CAR) study, an investigation of the diagnosis and assessment of AOD-related disorders conducted jointly by the World Health Organization (WHO) and the U.S. National Institute on Alcohol Abuse and Alcoholism (NIAAA) in nine countries between 1991 and 1993. Respondents participating in that study included heavy users of alcoholic beverages or certain other drugs, family members of heavy users, health workers in the area of AOD problems, and other health or social service workers having regular contact with people with AOD problems. In addition, each of the three countries was discussed in the *International Handbook on Alcohol and Culture* (Heath 1995) and in the *Alcohol and Emerging Markets* volume (Grant 1998). Finally, several ethnographic, epidemiological, and clinical studies have focused on drinking and its consequences in India, Mexico, and Nigeria. As a result, a considerable amount of current information is available on the drinking practices, drinking-related problems, and strategies for handling those problems in each of the countries studied (see also table 2).

India, Mexico, and Nigeria are all highly diverse nations with rich cultural variability with respect to ethnic, religious, and linguistic groups. The drinking patterns observed in the countries reflect that variability, and results obtained in specific regions of each country can be generalized only to a limited extent. For example, one cannot accurately characterize the drinking patterns of all Indian ethnic and cultural groups based on findings for just one of those groups. Accordingly, researchers describing drinking practices in each country must specify the regions in which those practices were observed.

In this article, the information presented on Indian drinking practices is based on investigations conducted in Bangalore, a city in the southwestern state of Karnataka that served as a CAR study site. In addition to English, the primary native language spoken in that region is Kannada. With respect to their religious affiliation, most people in the Bangalore region are Hindu, although there are also substantial numbers of Muslims, Christians, Buddhists, Sikhs, and Jains (i.e., followers of an ascetic religious tradition founded in the sixth century B.C.). Additional information on Indian drinking practices in this article is based on two ethnographic studies of alcohol consumption conducted among the Rajput, a military caste in northwestern India (Castairs 1979; Dorschner 1983).

The information on Mexican drinking practices presented in this article was obtained primarily in the capital of Mexico City and its environs, which was also a CAR study site. The predominant language spoken in Mexico City is Spanish; the religion most commonly practiced is Roman Catholicism. Although the CAR study focused mostly on Mexico City, it also addressed some urban-rural differences. Additional information in this article is based on research conducted in Chiapas, a predominantly rural region (Eber 1995).

Much of the information on Nigerian drinking practices was obtained in the city of Ibadan in the southwestern part of the country, which also participated in the CAR study. The predominant native language spoken in that region is Yoruba, along with English, and the most common religious affiliation is Christianity. Additional information on Nigerian drinking practices was drawn from a study conducted among the Kofyar in central Nigeria (Netting 1979) and from two studies of drinking patterns among college students in various urban areas of Nigeria (Enekwechi 1996; Odejide et al. 1987).

For the purpose of establishing a general measure of the role that alcohol consumption plays in a culture or country, researchers commonly use the terms “wet” and “dry.” Wet cultures are those in which alcohol consumption is a highly valued part of social life. Conversely, in dry cultures, alcohol consumption typically is considered aberrant behavior (Bennett et al. 1993).

India, Mexico, and Nigeria differ in their wet-dry classification (Room et al. 1996). For example, compared with societies that are traditionally thought of as wet (e.g., Greece), India can be considered a dry culture. Nigeria and Mexico, in contrast, can be classified as intermediate (i.e., neither clearly wet nor clearly dry). In fact, Natera Rey (1995) suggested that Mexico was best considered a “mixed” society in that neither the dry nor wet categories neatly apply in terms of per capita consumption, characteristic patterns of consumption, or social consequences of drinking. More recently, however, Medina-Mora (1998) characterized Mexico as a “good example of a dry culture where the rate of abstainers is high, the dominant pattern of heavy drinking is rarely very heavy and binge drinking is common. Although it is integrated in everyday life, alcohol is at the same time seen as dangerous and harmful” (p. 279).

## Comparison of Alcoholic Beverage Preferences

### India

In Bangalore the following three types of alcoholic beverages are consumed most often:

- *Arrack*, a traditional drink produced (both legally and illegally) by distilling fermented molasses, raw brown sugar, palm wine, rice, or palm sugar; it has an alcohol content ranging from 20 to 40 percent
- Palm wine, another traditional beverage produced from either the coconut tree or other palm trees, which has an alcohol content ranging from 20 to 40 percent
- Imported liquors, such as whiskey, brandy, and rum.

Beer is also consumed in the Bangalore region, although less commonly than the three types of beverages listed above. Of these alcoholic beverages, palm wine and beer are considered “cool” or “soft” drinks, whereas hard liquors and *arrack* are considered “hot” or “hard” drinks.

Studies among the Rajputs of northwestern India identified three preferred types of alcoholic beverages^1^ (Dorschner 1983):

- *Daru*, a drink distilled from the flowers of the mahwa tree and which ranges in alcohol content from 20 to 40 percent. At the time of the study, *daru* was considered inexpensive and was the most popular beverage in the Khaalapur community.
- Spirit produced from solvents, which greatly varies in alcohol content and, at the time of the study, was drunk only by “untouchables” and members of other lower castes.
- “English alcohol,” a distilled liquor—usually whiskey or gin—associated with British rule. At the time of the study, which coincided with a period of prohibition, English alcohol was extremely difficult to obtain.

The consumption of alcoholic beverages in India predates British colonization. However, in contrast to other countries—such as Nigeria and, to some extent, Mexico—alcohol consumption was not considered central to normal social life or daily meals in precolonial India. Nevertheless, in certain tribal groups throughout the country alcoholic beverages still are considered “a gift to humankind and, in turn, were reverently offered to the nature gods and other sacred powers” (Mohan and Sharma 1995, p. 134).

### Mexico

In Mexico, a wide variety of alcoholic beverages are consumed, including some that predate colonial settlement. Overall, however, Mexicans currently prefer beer and distilled alcoholic beverages, such as tequila and rum. In fact, beer accounted for 89 percent of all alcoholic beverages consumed in Mexico in 1989 (Natera Rey 1995). *Pulque*, wine, and 96-percent proof alcohol are the next favorite beverages. *Pulque*, a traditional drink with an alcohol content of 6 percent, is produced by fermenting a sugary liquid from the heart of a certain species of agave (i.e., the maguey plant). Like 96-percent proof alcohol, *pulque* is consumed primarily in rural parts of Mexico, whereas distilled beverages and wine are more popular in urban areas.

Some differences exist in the alcoholic beverage preferences of various population subgroups. For example, although both men and women drink *pulque*, women tend to prefer wine. Also, young people consume beer and wine more frequently than other alcoholic beverages (Medina-Mora et al. 1988).

Both drinking and intoxication occurred in Mexico before Spanish contact, but according to Natera Rey (1995):

Legitimate drinking was mostly ceremonial, confined to the upper classes, the old, and the wise. Commoners were allowed to drink only in certain situations: women after giving birth, to ‘strengthen their blood’; men and youths after exhausting work, to ‘restore their strength.’ Ceremonial drinking occurred as offerings to honor or placate the gods, to bless gatherings and collective tasks, to signal calendrical cycles, and promote fertility in the fields (p. 179).

Today *mezcal* (an intoxicating drink with an alcohol content of 40 percent that is distilled from the agave plant) and tequila are considered national alcoholic beverages in Mexico. However, these beverages were not produced before the Spanish conquest and the introduction of distillation techniques. For this reason, *pulque* is the alcoholic beverage most strongly associated with traditional Mexican rituals and customs.

### Nigeria

In the Ibadan region of Nigeria the most commonly consumed alcoholic beverages are palm wine, which is produced from the sap of the palm tree and has an alcohol content of 3 to 6 percent, and beer. Another typical alcoholic beverage consumed in that region is *burukutu*, which is fermented from guinea-corn and also ranges in alcohol content from 3 to 6 percent. Throughout Nigeria, native gin distilled from palm wine is popular (Oshodin 1995), as is beer. In particular, the Kofyar in central Nigeria favor beer. According to Netting (1979), “[t]he Kofyar make, drink, talk, and think about beer. It is the focus of cultural concern and activity” (p. 352). Alcohol consumption predates colonial rule in Nigeria; and, as in many African countries, alcoholic beverages are actually considered a type of food.

## Comparison of Epidemiological Patterns and Gender and Age Differences in Alcohol Consumption

### India

Few general population studies of alcohol consumption patterns have been conducted in India, and those that do exist were conducted primarily in the late 1970s, shortly after prohibition policies by the federal government and individual states were reversed. Furthermore, although various epidemiological studies have been conducted in specific regions of India, their generalizability to the entire country is questionable, at least in part because of methodological problems (Isaac 1998). The most consistent finding in all of the studies was that men are the primary consumers of alcoholic beverages. However, the percentage of men who had consumed an alcoholic beverage in the previous year varied widely among different regions, ranging from 16.7 percent in Madras City in southern India to 49.6 percent in a Punjab village in northwest India (Isaac 1998). Conversely, the alcohol consumption rates among women were consistently low (i.e., less than 5 percent) (Isaac 1998).

In a study of a clan of Rajput in the city of Khaalapur, Dorschner (1983) identified three types of drinkers: abstainers, social drinkers, and alcoholics. Of the men included in the study, 44.5 percent were abstainers, 16.4 percent were social drinkers, and 39.1 percent were heavy drinkers or alcoholics. Thus, although slightly more than one-half of the men consumed *daru*, spirit, or “English alcohol,” moderate social drinking was not the norm among those drinkers. In contrast, all the women in the study were abstinent. These overall drinking patterns are consistent with national epidemiological studies conducted in India but contrast sharply with those of Asian countries and many Western countries.

One reason for the specific drinking patterns in India may be the strong advocacy of abstinence by Indian religious groups. For example, among the Hindus (who make up more than 80 percent of India’s population) alcoholic beverages are forbidden for Brahmins and other upper-caste groups who are strict vegetarians. Members of all other caste groups who are meat eaters (e.g., the warrior, farmer, and scavenger-untouchable castes) are permitted to drink. Muslims also are not supposed to drink, although some Muslim men consume alcohol. Finally, Buddhists and Jains, who are strict vegetarians, are forbidden to drink. In addition, political prohibition policies in certain states may contribute to Indian drinking patterns.

Another epidemiological study conducted in the rural areas of Rajasthan demonstrated that although alcohol consumption had become accepted among men, it was still infrequent among women (Sundaram et al. 1984). However, drinking by women was more accepted in certain castes, particularly on festive occasions, such as weddings (Sundaram et al. 1984). In general, by the early 1980s alcohol consumption apparently had become an accepted leisure activity for men who were married and living in small families (i.e., husband, wife, and no more than three children) in this rural area (Sundaram et al. 1984). Furthermore, alcohol consumption rates were substantially higher among Hindus than among either Muslims or Jains, who rarely consumed alcoholic beverages.

### Mexico

Compared with other countries, Mexico has a relatively low per capita consumption of alcoholic beverages. In 1984 people older than age 15 consumed an average of 5.4 liters of pure alcohol^2^ (Medina-Mora et al. 1988). In another epidemiological study, the consumption of pure alcohol among people ages 15 and older ranged from 4.3 to 4.9 liters between 1972 and 1984 (Campillo et al. 1987). These relatively low numbers are somewhat misleading, however, because they are based solely on legally produced alcoholic beverages and do not include the large quantity of illegally produced alcohol consumed (Campillo et al. 1988).

Another confounding factor in interpreting the relatively low per capita alcohol consumption in Mexico is that a high proportion of Mexicans do not drink. For example, according to the National Survey of Addiction conducted in 1988 among people ages 18 to 65 living in urban areas of Mexico, 46.5 percent of the people in that age group abstained from alcohol consumption (i.e., drank an alcoholic beverage only once per year or less) (Natera Rey 1995). Abstinence rates differed substantially between men and women (26.6 percent versus 63.3 percent, respectively). However, those people who did drink—primarily men—tended to drink frequently and, in many cases, heavily. In fact, only a small proportion of the Mexican population was responsible for most of the alcohol consumption: 75 percent of the available alcohol was consumed by only 25 percent of the drinkers (Natera Rey 1995).

Several studies have investigated drinking patterns among Mexicans. In a 1989 survey of the adult population of Mexico City, only 17 percent of adults reported drinking an alcoholic beverage at least once per week (Medina-Mora 1998). However, 31 percent of respondents reported being intoxicated at least once in the preceding year. These findings may indicate that “the European pattern of frequent consumption of low quantities of alcohol does not seem to characterize the culture” (Medina-Mora 1998, p. 273). The majority of regular drinkers are men, whereas women who consume alcohol are generally infrequent drinkers. Overall, men drink more often and consume greater amounts of alcohol than do women. Among both Mexican men and women, the prevalence of drinking increases with increasing income but decreases with increasing age (Medina-Mora et al. 1988).

Differences in drinking patterns between rural and urban areas also have received considerable attention. According to some studies, alcohol consumption is substantially higher in certain rural areas. For example, in certain parts of Mexico a considerable amount of drinking occurs in connection with the celebration of numerous festivals. According to Natera Rey (1995), “ancestral custom mixed with modern elements” characterize alcohol consumption patterns in both Indian communities and rural areas of Mexico (pp. 184–185).

### Nigeria

Studies conducted in the 1970s on alcohol use in Nigeria focused primarily on the drinking practices of middle-aged and young adult men. In recent years, however, younger students (i.e., students between approximately 12 and 14 years of age who are in the early grades of secondary school) have become more involved in drinking. Specifically, since 1977 both availability and consumption of alcoholic beverages have increased substantially among young Nigerians, particularly secondary school students.^3^ To evaluate the extent and effect of these changes, Odejide and colleagues (1987) conducted a study of the drinking practices among secondary school students approximately 16 years old in Ibadan and Abeokuta, two major cities located in western Nigeria. Of the two cities, Ibadan is more industrialized. The study yielded the following results:

- Alcohol consumption among the students was significantly higher in Ibadan than in Abeokuta.
- Palm wine was the preferred drink in both cities.
- In both cities, alcohol consumption occurred significantly more often among students from middle- and upper-class families than among students from lower-class families.
- Female students overall drank less often than male students. Moreover, when female students did drink, it was typically at family festivities. Conversely, male students drank more regularly and at daily and weekend activities as well as on special occasions.
- In Ibadan, the majority of students had tasted alcoholic beverages—primarily palm wine—by age 11.

In another study conducted among students at the University of Nigeria, Enekwechi (1996) noted significant differences between men and women with respect to influences on their drinking. For example, women reported a greater need to consume alcoholic beverages at parties, whereas men reported that they were influenced by their parents’ drinking and by the desire to be accepted by their friends. Furthermore, men believed more strongly than women that alcoholic beverages helped enhance their sexual performance and calm their nerves. Enekwechi (1996) concluded that “males are more likely to drink for psychological and social reasons than females” (p. 5).

## Comparison of Typical Drinking Contexts and Preferred Drinking Patterns

### India

Researchers often use generalized categories to characterize drinking patterns in different countries. Such categories include abstinent cultures, such as those typically found in Muslim societies; permissive drinking cultures that are characteristic of many Mediterranean countries; overly permissive cultures (e.g., Japan and France); and ambivalent cultures, such as those of many Scandinavian and English-speaking cultures. India does not conform to any of these categories (Mohan and Sharma 1995). Instead, Indian attitudes about drinking include both permissive and abstinent features, especially when different population groups are considered.

This variability is not surprising given the broad religious, ethnic, and caste differences found in India (Mohan and Sharma 1995). For example, in his ethnographic study conducted in Rajasthan, Castairs (1979) compared caste differences in the use and significance of the traditional beverage, *daru*. According to that study, men from the Rajput caste—hereditary fighters and landowners—drank *daru*, whereas Brahmins—members of the highest-ranking caste who are religious leaders—were opposed to *daru* consumption. In fact, Castairs (1979) stated that “[c]ertainly no Hindu who had tasted or even touched *daru* would enter one of his temples (not even a goddess temple) without first having a purificatory bath and change of clothes” (p. 298). Thus, the Brahmins, who represented the spiritual aristocracy, and the Rajputs, who represented the warrior and landowner aristocracy, each had their own privileges. For example, the Brahmins had the right to hold intellectual positions in government or the private sector. However, they were vegetarians and were forbidden to drink alcohol. Conversely, the Rajputs’ privileges included the right to drink alcohol and eat meat, and many Rajputs prided themselves on a regular, moderate drinking pattern.

Even under British rule and with the introduction of English drinking customs, Indians generally did not incorporate alcohol consumption into their regular social activities. Furthermore, drinking did not become a ritualized component of religion, as it had in many other cultures (Mohan and Sharma 1995). Beginning in 1947, when India became politically independent, a strong movement against alcoholic beverage distribution and consumption developed throughout the country. Indian states were given the prerogative to set their own temperance laws, and two states—Bombay and Madras—introduced abstinence laws by 1951. Other states later passed similar laws. Beginning around 1966, however, a shift in Indian alcohol policy occurred, leading to the end of prohibition at both the federal and state levels by 1976. As a result, beer production increased steadily throughout the 1980s (Mohan and Sharma 1995).

One approach to assessing and comparing attitudes about alcohol consumption in various countries is to determine people’s definitions of “normal” and “harmful” drinking as well as their perceptions of alcohol’s effects. In Bangalore, normal drinking generally was described as the consumption of small quantities of alcohol that did not interfere with a person’s responsibilities. Moreover, normal drinking occurred only on special occasions and not more than once or twice per week. Conversely, harmful drinking was defined as consuming *arrack* or hard liquor frequently and to such an extent that alcohol consumption led to personal and/or work problems.

People in the Bangalore region expected normal drinking to reduce unpleasant feelings (e.g., pain, pressure, anxiety, fatigue, and boredom); improve their mood; and provide a “kick” that would lead to a sense of euphoria, increased activity, and a lack of inhibitions. Conversely, people who had “quite a lot” to drink were described as being *mathu* (i.e., drunk, with a negative connotation) or *masthi* (i.e., drunk and experiencing negative consequences), as losing control of their behavior, and as experiencing a loss of consciousness (Bennett et al. 1993).

### Mexico

In contemporary Mexican society, in both urban and rural areas, alcohol consumption is a facet of all aspects of social and family life. Alcohol consumption is evident at all stages of the life cycle, from birth and baptism to weddings and funerals. Saints’ days, community celebrations, and other special occasions are marked by drinking (Natera Rey 1995). Nevertheless, a double standard exists with respect to drinking (Medina-Mora 1998). Thus, drinking by women is considered inappropriate, whereas drunkenness among men is tolerated, at least to a certain degree. In fact, men are expected to drink, and the ability to drink large quantities is considered “macho” (Natera Rey 1995).

The complex and pervasive role of alcohol consumption in religious and community rituals presents many obstacles to recovery from alcohol problems. For example, an ethnographic study among people in the Chiapas province demonstrated that it was exceedingly difficult for both men and women to recover from their own drinking problems or from the effects of a family member’s drinking problems (Eber 1995). The study also found that women used creative approaches to avoid developing drinking problems, such as participating in religious groups and cooperatives.

Although alcohol’s role in Mexican society differs substantially from its role in Indian society, the expectations of alcohol’s effects are similar. People in Mexico City expected normal drinking to enhance feelings of well being and happiness, provide relaxation (without, however, resulting in disinhibition), and help achieve freedom without anxiety. People who drank “quite a lot,” however, were described as being drunk, losing control, and becoming a “boozer” (Bennett et al. 1993).

### Nigeria

Alcohol consumption is a central feature of adult (i.e., age 18 and older) life in Nigeria and plays a major role in social, religious, political, and economic relationships. For example, alcoholic beverages can be given as gifts to a bride’s relatives and as part of the “bride price.” Alcoholic beverages are consumed at virtually all ceremonies, including festivals, weddings, and funerals. Moreover, drinking typically indicates hospitality. Although different age groups and men and women are not explicitly separated during these occasions, elders and men are expected to drink more than either younger people or women (Oshodin 1995).

People in Ibadan defined normal drinking as having a few drinks occasionally at social events or drinking in moderation to relax. Conversely, harmful alcohol use was described as excessive drinking that results in intoxication and in physical and social impairment. Nigerians generally considered it undesirable to drink too much (Oshodin 1995). The anticipated effects of normal drinking in Ibadan included relief of stress and inhibition as well as feelings of sociability, euphoria, and boldness. Although normal drinking was described as reinvigorating, however, it was also considered a cause of hyperactivity and sickness. Once a person had consumed “quite a lot” to drink, however, he or she was described as being uninhibited, sick, disoriented, irritable, voluble, clumsy, sleepy, or more alert or as behaving irresponsibly^4^ (Bennett et al. 1993).

## Comparison of Alcohol-Related Problems and Approaches to Prevention and Treatment

### India

Although alcohol consumption is not an integral part of Indian social life, alcohol-related problems can occur. For example, in one study conducted in Rajasthan, 24.7 percent of all people age 15 and older (36.1 percent of men and 13.4 percent of women) consumed alcohol. Furthermore, 3 percent of people in that age group (5.6 percent of men and 0.5 percent of women) were considered alcohol dependent (Sundaram et al. 1984). Alcohol-related problems do not affect all population subgroups equally, however, because in the same study, Hindus were more likely to drink than were Jains and Muslims. Overall, a growing number of health, social, and economic problems attributable to rising alcohol consumption have been documented throughout India (Isaac 1998). Treatment facilities for these problems are organized by governmental or nongovernmental (both private and public) organizations.

Policies to control alcohol production and consumption in India were initiated after the country’s independence in 1947. For example, article 47 in the Indian constitution states that “[t]he state shall endeavor to bring about prohibition of the consumption except for medicinal purposes of intoxicating drinks and drugs which are injurious to health” (Isaac 1998, p. 148). Between the mid-1960s and 1976, however, the national government’s commitment to total prohibition ceased. As a result, the production and sale of alcoholic beverages were greatly simplified during the 1980s and 1990s, and shops, bars, and restaurants now can easily obtain a license to sell alcoholic beverages. In fact, national policies regarding alcoholic beverages have “swung from total prohibition to unrestricted sale with no controls” (Isaac 1998, p. 151).

Most Indian state governments also are ambivalent about prohibition (Isaac 1998). Although many states have established temperance boards for educating Indian people about the potentially harmful effects of drinking, these boards primarily sponsor newspaper advertisements.

### Mexico

Alcohol-related problems are reportedly common among Mexican men (Medina-Mora 1998) and may have a substantial adverse effect on the overall health and well being of the Mexican population (Campillo et al. 1988). During the 1970s and 1980s, Mexico had one of the highest mortality rates from liver cirrhosis in Latin America (i.e., 20 to 23 deaths among 100,000 people). Furthermore, alcohol may be associated with most absences from work (Campillo et al. 1987).

In 1982 the Mexican Ministry of Health assumed control of and responsibility for the nation’s health policies. This move has affected policies and programs addressing AOD-related problems throughout the country. Beginning in 1983, the National Council Against Addictions initiated measures to prevent alcohol-related problems in Mexico. As a result of those initiatives, by 1988, 16 of the 31 Mexican states had signed measures such as forbidding the sale of alcoholic beverages to children younger than age 18 and to people who were intoxicated. Since then, the council’s work has continued and is currently under review, with an understanding that “[m]ore emphasis needs to be placed on local problems, along with the development of specific subprograms” (Medina-Mora 1998, p. 281). Other measures introduced in recent years include educational programs, warning labels, an increase in the price of pure cane alcohol, training in medical schools, and early identification and treatment programs (Campillo et al. 1988). A particular emphasis has been placed on early detection, especially among young and middle-aged men, the two population subgroups at the greatest risk for alcohol-related problems (Campillo et al. 1987).

Mexican women appear to be less likely to receive professional treatment for alcohol-related problems. Thus, according to the National Survey on Addictions, a notable difference exists between the ratio of men to women attending Alcoholics Anonymous (AA) programs (i.e., 10:1) and the ratio of men to women in formal treatment programs (i.e., 16.7:1) (Medina-Mora 1994). This difference indicates that women are substantially less likely to enter formal treatment than to choose an informal treatment approach, such as AA. Furthermore, special interventions targeted at women are not available in either formal treatment programs or AA, even though women are expected to control their own drinking as well as that of the men in their families (Medina-Mora 1994).

### Nigeria

Alcoholic beverages are the most widely abused psychoactive substances in Nigeria, and the Nigerian government has recognized the need to establish policies to control both alcohol production and consumption. In 1920 measures were established to control the importation, sale, and local fermentation and distillation of alcohol, including the requirement for a special permit (Oshodin 1995). Because drinking is an integral part of daily and ceremonial life of those Nigerians who are not Muslims, however, government efforts to establish prevention and treatment programs have had little effect. Compared with many other countries, the Nigerian government is not very strict in implementing policies regulating alcohol production, distribution, and consumption. For example, although existing laws regulate when and where alcohol can be sold, they are not strictly enforced. In addition, no age restrictions on the purchase or consumption of alcoholic beverages exist (Oshodin 1995).

The position of Nigerian women in regard to alcohol-related problems is particularly difficult (e.g., Ikuesan 1994). In most rural areas, women are strongly involved in the production of alcoholic beverages, which may promote drinking. Also, it generally is considered acceptable for women to drink (except among Muslim Nigerians). Drinking problems among women, however, cause great disruption to their families and result in stigmatization by the community (Ikuesan 1994).

## Comparison of Drinking Indicators

To gain a better understanding of what is considered “normal” drinking or alcohol dependence in various cultures, researchers can assess and compare characteristics that are considered signs of intoxication or an indication that a drinker requires medical attention. In the CAR study, those indicators were relatively similar among the Indian, Mexican, and Nigerian study sites, although some differences existed. At all three sites, becoming aggressive and being uncoordinated were considered signs of intoxication. With respect to indicators of the need for medical attention, the following indicators were considered critical:

- In Bangalore: head injuries or unconsciousness
- In Mexico City: loss of consciousness, violence, and physical injuries
- In Ibadan: sustained injuries, persistent vomiting, and unconsciousness.

Thus, at each site, medical attention was not considered necessary unless a serious disturbance of the central nervous system (i.e., loss of consciousness) and alcohol-related bodily injuries occurred (Room et al. 1996).

In contrast to the similarities among the three sites in the indicators of intoxication or need for medical attention, the thresholds for a diagnosis of alcohol dependence or addiction as defined by heavy drinkers, their family members, and health care professionals differed widely. For example, in Bangalore, where drinking occurs among a minority of men and is rare among women, respondents indicated extremely low thresholds for a dependence diagnosis. Thus, one person stated that “drinking the equivalent of three bottles of beer 1–3 days a month, and never drinking more; objections from friends, doctor, or clergy; trouble driving because of drinking; the same amount of alcohol having less effect than before; and having had such a strong desire to . . . drink that he could not resist it” were indicative of dependence (Room et al. 1996, p. 217). Substantially higher levels of alcohol consumption were considered necessary for a diagnosis of alcohol dependence in both Mexico City and Ibadan.

Another measure of people’s attitudes regarding alcohol is their perception of who or what is responsible for a person’s drinking (e.g., the drinker; the alcoholic beverage; or external factors, such as social and economic pressure). In Bangalore, respondents considered the alcoholic beverage primarily responsible for intoxicated behavior and thought that drinkers had relatively little control over their own behavior. Furthermore, heavy drinking was not expected to produce any positive effects.

People in Mexico City had no clear-cut opinion as to whether the alcoholic beverage or the drinker was responsible for intoxicated behavior. In addition, Mexicans thought that both the drinker’s self-control and social pressures influenced a person’s drinking behavior. People who were alcoholics, however, were no longer considered able to control their drinking. Mexican respondents, like those in Bangalore, saw no positive effects resulting from heavy drinking.

Finally, respondents in Ibadan did not consider drinkers to be responsible for changes in their behavior when they were intoxicated. Moreover, various factors, such as social situations, economic factors, and other people, were perceived to have a major influence on a person’s drinking. As in Bangalore and Mexico City, heavy drinking was not expected to have any positive effects.

## Conclusions

The studies summarized in this article demonstrate that drinking practices and the definition of what constitutes normal drinking vary sharply among different countries. Even within a country, substantial differences in these definitions and practices may exist among members of different ethnic or cultural groups. For example, although India generally is considered a dry country, drinking practices differ considerably between people living in the southern or northern areas, and even among members of different castes residing in the same region. As a result of the cultural and ethnic variations in drinking practices, no single definition of problem drinking or alcohol dependence exists that is uniformly applicable in all countries or cultures. Similarly, researchers, clinicians, and public health officials attempting to develop effective prevention and treatment approaches must consider the population’s attitudes and expectations regarding alcohol consumption (e.g., what constitutes heavy drinking) and its effects (e.g., whether the drinker or the alcoholic beverage is responsible for intoxicated behavior).

Cultures or countries such as the ones described in this article can undergo broad social and related changes, which typically are accompanied by alterations in drinking patterns. An example of a notable change in drinking patterns is the experience of Mexican men who migrate to the United States. According to Medina-Mora and colleagues (1988, p. 30), these migrants “modify their consumption patterns by adopting the more frequent drinking patterns preferred by Americans, maintaining, however, the high quantities per occasion common in Mexico.”

Changes in drinking practices often also influence the particular nature of alcohol-related problems and appropriate approaches to prevention and treatment in a culture. For example, examination of the differences in drinking practices in the three countries discussed in this article suggests that a focus on women’s drinking more likely would be pertinent in Mexico and Nigeria than in India, where women generally do not drink. Such a focus, in turn, could lead to the development of prevention and treatment programs tailored to the needs of women.

Although this article has focused primarily on cross-cultural variations among the three countries discussed, it is important to recognize the cultural variation within each of these nations. For example, although India’s prohibition policies of the past “have failed miserably” (Isaac 1998, p. 170), that country is in the midst of developing a master plan for reducing the demand for and supply of all psychoactive substances, including alcoholic beverages. However, India is characterized by tremendous cultural variability with respect to beliefs and practices regarding alcoholic beverage consumption. Furthermore, the country is experiencing changes in the traditional family structure, a weakening of informal cultural and religious controls on alcoholic beverage consumption, and increasing contact with drinking practices outside Indian society. As a result, Indian policymakers must pay attention to these cultural variations in establishing appropriate prevention and treatment programs for the diverse segments of Indian society.

## Figures and Tables

**Table 1:** Cultural Characteristics of Regions in India, Mexico, and Nigeria That Participated in the Cross-Cultural Applicability Research Study

City and Country	Location	Primary Language(s)	Primary Religion(s)
Bangalore, India	South Asia	Kannada, Tamil, Telugu, and Hindi	Hinduism, Islam, Christianity, Sikhism, Buddhism, and Jainism
Mexico City, Mexico	North America	Spanish	Roman Catholicism
Ibadan, Nigeria	West Africa	Yoruba	Christianity, Islam

SOURCE: Bennett et al. 1993.

**Table 2:** Differences in Drinking Practices Among Sites in India, Mexico, and Nigeria That Participated in the Cross-Cultural Applicability Research Study

Drinking Practice	Country		
	India	Mexico	Nigeria
Most commonly consumed alcoholic beverages	*Arrack*, palm wine, beer, and imported liquors	Beer, tequila, rum, 96-percent proof alcohol, *pulque*, wine, and *mezcal*	Palm wine and beer
Drinking predates European contact	Yes, but not particularly central to social life	Yes, especially *pulque* consumption as part of traditional rituals and customs	Yes, drinking (especially of palm wine and home-brewed beer) was part of normal social and ceremonial life
Women’s drinking patterns	Predominantly (about 95%) abstinent	Majority (about 63%) abstinent	Minority (about 30%) abstinent in two urban areas studied
Men’s drinking patterns	Highly variable across regions	Those who drink tend to drink rather frequently and often heavily	Most drink on a regular basis
Context of drinking occasions	No regular context established; thus far, not a part of regular daily life or ritual occasions	Is an aspect of social family life passages, fiestas and, for some drinkers, part of daily life	A central feature of adult male life and in social, religious, and economic relationships
Evidence of concerns about young people’s drinking	Increasing	Definitely increasing	Has been a concern for the past two decades in some areas
Extent of major concerns about alcohol-related problems	Increasing problems seen in health, social, and economic areas	Considerable concern about drinking among men; drinking is seen as having major consequences for health and job performance	Although there are increasing concerns, generally they are not viewed as sufficiently serious to establish many prevention and treatment programs

## Footnotes

1 These preferences may have changed during the past 15 years.

2 For comparison, the per capita alcohol consumption among people age 14 and older in the United States in 1984 was approximately 10.0 liters (National Institute on Alcohol Abuse and Alcoholism 1997). Another study noted that the per capita alcoholic beverage consumption in Mexico is 2.9 times lower than that in Chile (Edwards et al. 1994).

3 One potential reason for the increase in alcohol availability may be the increase in the number of breweries in Nigeria in the 1970s.

4 As indicated by these descriptions, contradictory viewpoints exist among Nigerians about the effects of having “quite a lot” to drink (e.g., expecting it to make people either sleepy or more alert).
